# Sea surface temperature (SST) and SST anomaly (SSTA) datasets over the last four decades (1977–2016) during typhoon season (May to November) in the entire Global Ocean, North Pacific Ocean, Philippine Sea, South China sea, and Eastern China Sea

**DOI:** 10.1016/j.dib.2022.108646

**Published:** 2022-09-30

**Authors:** Ravi Shankar Pandey, Yuei-An Liou

**Affiliations:** a Center for Space and Remote Sensing Research, National Central University, No. 300, Jhongda Rd., Jhongli District, Taoyuan City 320317, Taiwan, R.O.C.; b National Museum of Marine Science and Technology, No. 367, Beining Rd., Zhongzheng Dist., Keelung City 202010, Taiwan, R.O.C.; c Institute of Earth Sciences, Academia Sinica, 128 Academia Road, Section II, Nankang, Taipei 11529, Taiwan, R.O.C.

**Keywords:** Sea surface temperature, North West Pacific, Sea surface temperature anomaly, Global ocean, North Pacific Ocean, Philippine Sea, South China Sea, Eastern China Sea

## Abstract

The Sea Surface Temperature Anomalies (SSTA) are created for the chosen study period from 1977 to 2016 (40-years) including the base period from 1941 to 1970 (30-years) using the two different raw Sea Surface Temperature (SST) datasets named Optimum Interpolation (OI) SST version 2 and Centennial in situ Observation-Based Estimates (COBE) SST version 2. The SSTA and SST are measured for each month from May to November (typhoon activity months in the North West Pacific) over the entire Global Ocean, especially focusing on the North Pacific Ocean; Philippine Sea; South China Sea; and Eastern China Sea (the marginal Seas of the North West Pacific Ocean). The OI-SST V2 dataset is directly accessed by the online link https://psl.noaa.gov/, which is made available by the Physical Sciences Laboratory (PSL) of the National Oceanic and Atmospheric Administration (NOAA). OI-SST V2 dataset contains monthly-averaged SST data from December 1981 to May 2020. COBE-SST V2 dataset belongs to the Japan Meteorological Agency (JMA) and is also made available by the PSL of NOAA through the online link https://psl.noaa.gov/. COBE-SST V2 dataset contains a very long period of monthly-averaged SST data from January 1850 to December 2019. The SST data in both datasets are on a regular one-degree (1^o^) grid covering the entire Oceans of the Earth. Both datasets are in the Network Common Data Form (NetCDF)(.nc) and can be opened on any appropriate software platform like ESRI ArcGIS 10.5 for further analysis. All SST data presented in this article merely belong to the typhoon season months (from May to November) of the North West Pacific (NWP) Ocean basin and are thus crucial for typhoon-related research. At First, the SST data for each month from May to November over the whole study and the base periods are extracted for the entire Global Ocean. Then, for each successive 5-year period and 10-year period, the SST data is averaged separately for each month from May to November. Also, for the whole 40 years of the chosen current period and 30 years of the base period, the SST data is averaged separately for each month of the typhoon season. The successive year, 5-year, and 10-year SST data of the chosen current period is averaged for all seven months of typhoon season. Also, for the whole 40 years of the chosen current period and 30 years of the base period, the SST data is averaged over all seven months of typhoon season. Finally, the yearly, 5-yearly, 10-yearly, and monthly Sea Surface Temperature Anomalies (SSTA) are measured using the chosen current and base period data for the entire Global Ocean, North Pacific Ocean, Philippine Sea, South China sea, and Eastern China Sea. Statistical analyses are done, which are significant for global warming, SST, and typhoon-related research. For detailed analysis, explanation, and discussion, the readers are referred to the “Typhoon strength rising in the past four decades” [1].

**Specifications Table**

**Table:** 

Subject	Atmospheric Science
Specific subject area	Temporal SST variations during 1977-2016 over the entire Global Ocean, North Pacific Ocean, Philippine Sea, South China sea, and Eastern China Sea
Type of data	Table, Figure, Chart
How data were acquired	The SST data were extracted for the desired zones and time from two different SST raw datasets and later converted to SST Anomaly. The raw dataset was collected from the Optimum Interpolation (OI) SST version 2 and Centennial in situ Observation-Based Estimates (COBE) SST version 2 public databases which were provided by the Physical Sciences Laboratory (PSL) of the National Oceanic and Atmospheric Administration (NOAA) (https://psl.noaa.gov/) [2,3]. Raw Data was sorted manually then processed in ArcGIS (ESRI ArcGIS 10.5) and collected in ms-word sheets.
Data format	Raw, Processed
Description of data collection	The dataset of SST anomaly is formed using 70-years of SST data, where the 30-years are used to create base periods and the rest 40-years for study. The data covers the whole global ocean especially focusing on the marginal Seas of the North West Pacific. The original SST archive data of the Optimum Interpolation (OI) SST version 2 and the Japan Meteorological Agency's (JMA) Centennial in situ Observation-Based Estimates (COBE)-SST version 2 data were directly accessed from the online link https://psl.noaa.gov/, which is made available by the Physical Sciences Laboratory (PSL) of the National Oceanic and Atmospheric Administration (NOAA). Both datasets are freely available in the public domain [2,3]. Few further analyses are done to measure Sea Surface Temperature Anomaly (SSTA) data.
Data source location	Institution: The Physical Sciences Laboratory (PSL) of the National Oceanic and Atmospheric Administration (NOAA). City/Town/Region: Boulder County, Colorado Country: United States of America Latitude and longitude (and GPS coordinates, if possible) for collected samples/data: 39.99°N, 105.26°W Institution: The Japan Meteorological Agency (JMA). City/Town/Region: Tokyo Country: Japan Latitude and longitude (and GPS coordinates, if possible) for collected samples/data: 35.6762° N, 139.6503° E The raw data is available through links https://psl.noaa.gov/data/gridded/data.noaa.oisst.v2.html[2] and https://psl.noaa.gov/data/gridded/data.cobe2.html[3].
Data accessibility	Repository name: Mendeley Data Data identification number: DOI:10.17632/4ynzr5n3s6.2 Direct URL to data: https://doi.org/10.17632/4ynzr5n3s6.2
Related research article	R.S. Pandey, Y.A. Liou, Typhoon strength rising in the past four decades, Weather and Climate Extremes. 31 (2022) 100446. https://doi.org/10.1016/j.wace.2022.100446[1]

**Value of the Data**

• This manuscript adds crucial information to its related published article. It provides Tables of extracted Sea Surface Temperature (SST) and measured Sea Surface Temperature Anomalies (SSTA) showing year-wise and month-wise variations during four decades of the study period, whereas in the related article only decadal and half-decadal averaged SSTA-based analysis was used. The SST anomalies are calculated using 70-year monthly SST data from two different sources and extracted only for the months when typhoons are active in the North West Pacific basin and were focused over all four marginal Seas, which are responsible for the cyclogenesis. Any kind of typhoon-related research needs such data where a clear impact of climate change is also visible by the gradual increase in SST and SSTA. Moreover, similar SST and SSTA measurements are also done for the whole Global Ocean, which can be useful for various researchers worldwide for various purposes. The manuscript provides far more crucial data and information, which are not introduced in the published article, and also the resembling things are removed.
• The SST and SST Anomaly data provided in this article supersedes the normal SST data as it concerned only the months of typhoon season (May-November) of the North West Pacific, which makes it important for typhoon-related research. Moreover, it extracts specifically the temporal SST variations in all major seas of the North West Pacific separately, for detailed information and possible use in various climate change-related research.
• The SST anomalies are created using 70-year data, among which 30-year data are used to form the base and 40-year data for further analysis. Each month from May to November is also analyzed separately.
• The SST data provided in this article also can be used to explore the temporal varying patterns of the SST over the global ocean as well as over the NWP Ocean basin (through the SST of its marginal seas like the North Pacific Ocean, Philippine Sea, South China sea, and Eastern China Sea) during the last 4 decades (1977-2016).
• The SST data provided in this article can be useful to study global warming and/or typhoons for related researchers worldwide as well as to the government agencies which are responsible for disaster risk assessment, mitigation, and preparedness.
• The SST and SSTA data provided in this article can be used as a base to further explore the physical mechanism behind the varying temporal SST or SSTA in the Global Ocean, North Pacific Ocean, Philippine Sea, South China sea, and Eastern China Sea.

## 1 Data Description

**Fig. 1:**
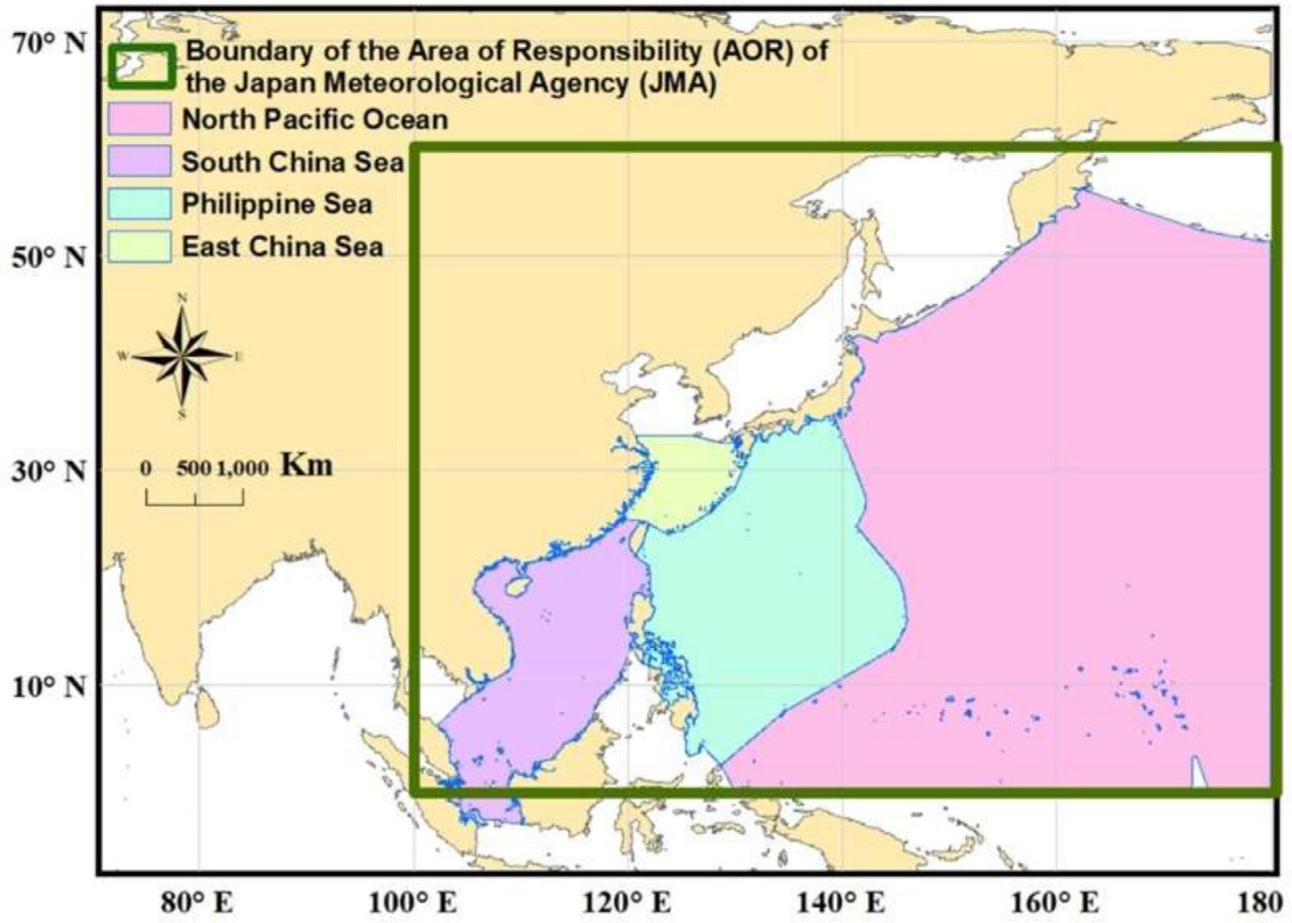
The Area of Responsibility (AOR) of the Japan Meteorological Agency (JMA) is in the small box.

The Sea Surface Temperature (SST) and SST Anomalies (SSTAs) are created for the four decades of the study period (1977-2016), where the base period is of three decades (1941-1970). The measurements are done for each month from May to November, which is considered the typhoon activity months in the North West Pacific. Although, the coverage is the whole global ocean but a special focus is kept on all four marginal seas of the North West Pacific Ocean named North Pacific Ocean, Philippine Sea, South China sea, and Eastern China Sea. The two different raw Sea Surface Temperature (SST) datasets are used for the purpose named Optimum Interpolation (OI) SST version 2 and Centennial in situ Observation-Based Estimates (COBE) SST version 2. The details about the SST raw datasets are provided below:

The Japan Meteorological Agency (Kishō-chō, JMA) is the Regional Specialized Meteorological Centre (RSMC) of Asia of the World Meteorological Organization (WMO) since 1968. The Tokyo Climate Center, of the Climate Prediction Division, which comes within the JMA is responsible for the Sea Surface Temperature (SST) analysis for climate monitoring and predictions. The center contains the long-term SST data from January 1850 to December 2019 with the east-west and north-south grid points. The east-west grid points run eastward from 0.5^o^ E to 0.5^o^ W, while the north-south grid points run northward from 89.5^o^ S to 89.5^o^ N, covering almost the entire sea surface of the earth. The SST dataset is known as Centennial in situ Observation-Based Estimates (COBE) SST which has an aerial resolution of 1^o^ latitude and 1^o^ longitude [4]. Recently, in March 2006, the method involved in creating the COBE-SST dataset is updated [5]. Whereas, the method of Folland and Parker (1995) was used for the analysis of past SST observation reports [6]. For the second version of the COBE-SST V2 dataset, a new SST analysis on a centennial time scale is used as described ahead [7]. COBE-SST V2 dataset is created by the use of in situ SST and sea-ice concentrations as a sum of the trend, interannual variations, and daily variations. Theory-based analysis errors are used as a measure of reliability. The ice-SST relationship is used by an improved equation to produce SST data from observed sea ice concentrations. The biases of individual SST measurement types are estimated for a homogenized long-term time series of global mean SST before the analysis to ensure consistency because the bias correction is unavailable for many historical observational reports. The resultant daily COBE-SST V2 fields are converted into monthly fields by averaging to provide monthly averaged SST with spatial coverage of 89.5° N – 89.5° S and 0.5° E – 359.5° E. Note here that the Area of Responsibility (AOR) of JMA, which covers the NWP and the South China Sea (0^o^–60^o^ N, 100^o^–180^o^ E) including marginal seas and adjacent land areas is the typhoon activity zone (Fig. 1). COBE-SST version 2 data were directly accessed from the online link https://psl.noaa.gov/, which is made available by the Physical Sciences Laboratory (PSL) of the National Oceanic and Atmospheric Administration (NOAA) [2,3].

The monthly Optimum Interpolation (OI) Sea Surface Temperature (SST) version-2 dataset belongs to the National Oceanic and Atmospheric Administration (NOAA). The dataset is made by a combination of satellite SST and in situ (Argo floats, ships, and buoys) data. It also includes the sea-ice cover simulated SST. It has a spatial resolution of 1^o^ × 1^o^ with a spatial coverage of 89.5° N – 89.5° S and 0.5° E – 359.5° E. The SST analysis is done by filling spatial gaps by interpolation. The bias adjustment of satellite and ship observations is done using the method of Reynolds to compensate for platform differences and sensor biases [8]. The linear interpolation and averaging of the daily values over a month are used to convert the weekly to daily OI-SST V2 fields into the monthly fields. The monthly OI-SST V2 dataset contains monthly averaged global SST from December 1981 to May 2020. The monthly OI-SST version 2 data were directly accessed from the online link https://psl.noaa.gov/, which is made available by the Physical Sciences Laboratory (PSL) of the National Oceanic and Atmospheric Administration (NOAA). Both above-mentioned SST datasets are used in a recent paper “Typhoon strength rising in the past four decades” [1].

### 1.1 Sea surface temperature data

**Fig. 2:**
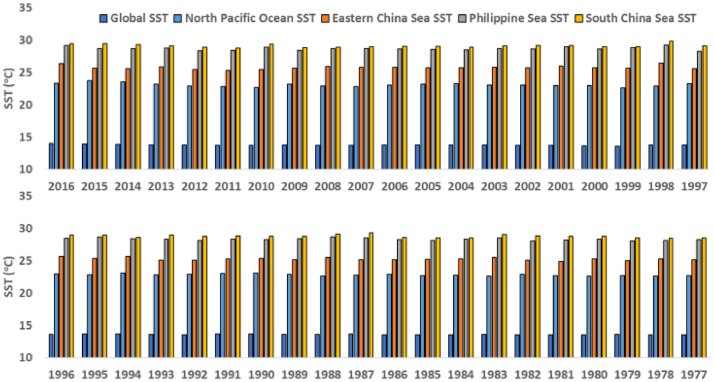
Chart of year-wise Sea Surface Temperature based on data of Table 1.

Table 1 provides the typhoon seasonally averaged SST data for each year of the current chosen period (1977-2016) and averaged over the typhoon season. All SST values are averaged over the typhoon season months of May to November. Here, GLO, ECS, PS, SCS, and NPO represent the Global Ocean, Eastern China Sea, South China Sea, and North Pacific Ocean, respectively. All SST values are in °C. Fig. 2 shows the year-wise SSTA data of Table 1 for easy understanding for readers.

**Table 1:** Yearly Sea Surface Temperature from 1977 to 2016.

Year	2016	2015	2014	2013	2012	2011	2010	2009	2008	2007	2006	2005	2004	2003	2002	2001	2000	1999	1998	1997
**GLO SST**	14.0	13.9	13.9	13.8	13.8	13.7	13.7	13.8	13.7	13.7	13.8	13.8	13.8	13.8	13.7	13.7	13.6	13.6	13.8	13.8
**ECS SST**	26.4	25.7	25.6	25.9	25.4	25.3	25.4	25.6	26.0	25.8	25.8	25.7	25.7	25.8	25.7	26.0	25.7	25.6	26.5	25.6
**PS SST**	29.2	28.7	28.7	28.7	28.4	28.4	28.9	28.4	28.7	28.7	28.6	28.5	28.5	28.7	28.6	29.0	28.6	28.8	29.2	28.3
**SCS SST**	29.4	29.4	29.3	29.1	28.8	28.7	29.4	28.8	28.9	28.9	29.0	29.0	28.9	29.1	29.2	29.2	29.0	29.0	29.8	29.1
**NPO SST**	23.3	23.7	23.5	23.2	22.9	22.9	22.7	23.2	22.9	22.8	23.0	23.1	23.2	23.1	23.0	22.9	22.9	22.6	22.9	23.2

Year	1996	1995	1994	1993	1992	1991	1990	1989	1988	1987	1986	1985	1984	1983	1982	1981	1980	1979	1978	1977

**GLO SST**	13.6	13.7	13.6	13.6	13.5	13.6	13.7	13.6	13.6	13.7	13.5	13.5	13.5	13.6	13.5	13.5	13.5	13.6	13.5	13.5
**ECS SST**	25.7	25.4	25.7	25.1	25.1	25.3	25.3	25.1	25.5	25.2	25.1	25.3	25.3	25.5	25.1	24.9	25.3	25.0	25.3	25.1
**PS SST**	28.5	28.7	28.4	28.3	28.2	28.3	28.3	28.4	28.7	28.5	28.3	28.1	28.4	28.5	28.0	28.2	28.4	28.1	28.2	28.2
**SCS SST**	29.0	29.0	28.6	28.9	28.7	28.8	28.8	28.7	29.1	29.3	28.6	28.5	28.5	29.0	28.8	28.7	28.7	28.6	28.5	28.5
**NPO SST**	22.9	22.8	23.1	22.8	22.9	23.0	23.1	22.9	22.6	22.8	22.9	22.7	22.7	22.6	22.9	22.7	22.6	22.7	22.6	22.7

**Fig. 3:**
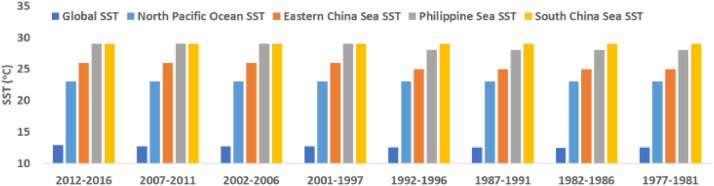
Chart of 5-yearly Sea Surface Temperature based on data of Table 2.

Table 2 provides the 5-yearly and typhoon seasonally averaged SST data from 1977 to 2016. All SST values are averaged over the typhoon season months of May to November. Mx, Mn, and MN represent maximum, minimum, and mean SST values, respectively. Fig. 3 shows the 5-yearly SST data of Table 2 for easy understanding for readers.

**Table 2:** 5-Yearly Sea Surface Temperature from 1977 to 2016.

5-yearly Averaged Sea Surface Temperature (°C) during the whole typhoon Season (May- November)																								
Time Period	2012-2016			2007-2011			2002-2006			2001-1997			1992-1996			1987-1991			1982-1986			1977-1981		
Ocean of Cyclogenesis	Mx	Mn	MN	Mx	Mn	MN	Mx	Mn	MN	Mx	Mn	MN	Mx	Mn	MN	Mx	Mn	MN	Mx	Mn	MN	Mx	Mn	MN
Eastern China Sea	28	22	26	28	22	26	28	22	26	28	22	26	28	22	25	28	21	25	28	21	25	28	20	25
Philippine Sea	30	24	29	30	24	29	29	25	29	30	25	29	30	24	28	29	24	28	29	24	28	29	24	28
South China Sea	30	26	29	30	26	29	30	26	29	30	26	29	30	26	29	30	26	29	30	26	29	29	26	29
North Pacific Ocean	30	07	23	30	07	23	30	07	23	30	07	23	30	07	23	30	06	23	30	06	23	29	06	23
**Global Ocean**	31.4	-1.8	12.9	31.5	-1.8	12.7	31.6	-1.8	12.7	31.7	-1.8	12.7	31.0	-1.8	12.6	31.0	-1.8	12.6	30.7	-1.8	12.5	31.2	-1.8	12.6

**Fig. 4:**
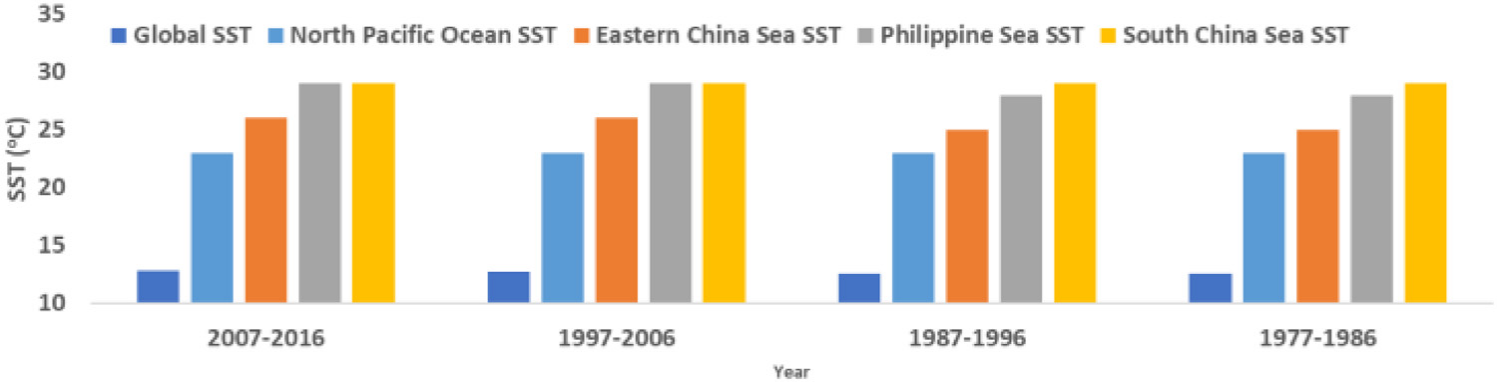
Chart of decadal Sea Surface Temperature data based on data of Table 3.

Table 3 provides the 10-yearly (decadal) and typhoon seasonally averaged SST data from 1977 to 2016. The table also provides SST data for the whole chosen 40 years. All SST values are averaged over the typhoon season months of May to November. Mx, Mn, and MN represent maximum, minimum, and mean SST values, respectively. Fig. 4 shows the decadal SST data of Table 3 for easy understanding for readers.

**Table 3:** 10-Yearly and 40-years Sea Surface Temperature from 1977 to 2016.

Decadal Averaged Sea Surface Temperature (°C) during the whole typhoon Season (May- November)															
Time Period	2007-2016			1997-2006			1987-1996			1977-1986			1977-2016		
Ocean of Cyclogenesis	Mx	Mn	MN	Mx	Mn	MN	Mx	Mn	MN	Mx	Mn	MN	Mx	Mn	MN
Eastern China Sea	28	22	26	28	22	26	28	21	25	28	21	25	28	22	26
Philippine Sea	30	24	29	30	25	29	29	24	28	29	24	28	29	24	29
South China Sea	30	26	29	30	26	29	30	26	29	30	26	29	30	26	29
North Pacific Ocean	30	07	23	30	07	23	30	07	23	29	06	23	30	07	23
Global Ocean	31.5	-1.8	12.8	31.6	-1.8	12.7	30.9	-1.8	12.6	30.9	-1.8	12.6	31.2	-1.8	12.7

### 1.2 Sea Surface Temperature Anomaly Data

**Fig. 5:**
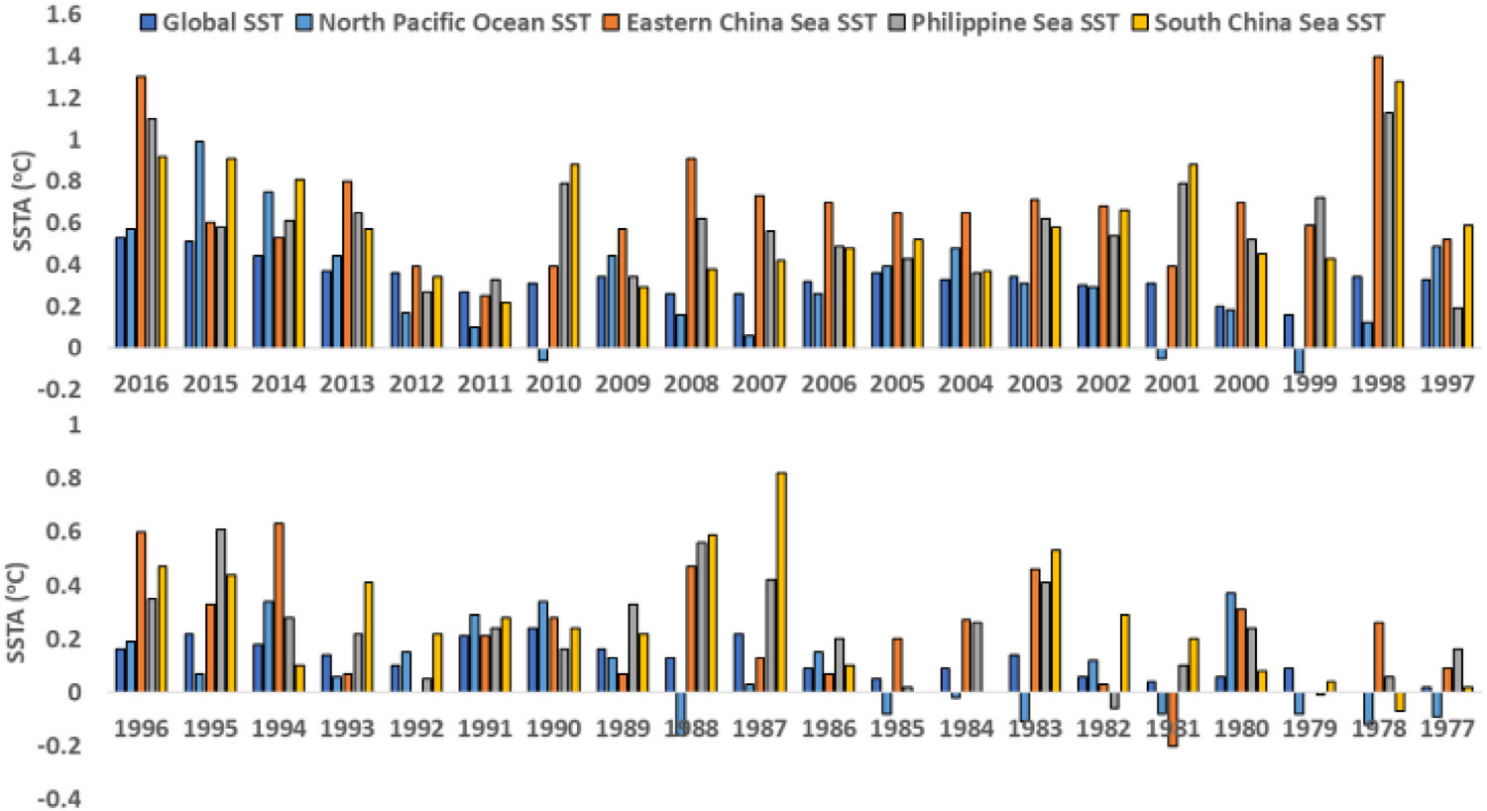
Chart of year-wise Sea Surface Temperature Anomaly based on data of Table 4.

Table 4 provides the typhoon seasonally averaged SSTA data for each year of the current chosen period (1977-2016) and averaged over the typhoon season. The base years are 1941-1970. Note here that all 30-years of base period averaged over typhoon season has maximum, minimum, and average SST values as 31.21°C, -1.88°C, and 13.38°C, respectively, for the whole Global Ocean. All SSTA values are averaged over the typhoon season months of May to November. Here, GLO, ECS, PS, SCS, and NPO represent the Global Ocean, Eastern China Sea, South China Sea, and North Pacific Ocean, respectively. All SSTA values are in °C. Fig. 5 shows the year-wise SSTA data of Table 4 for easy understanding for readers.

**Table 4:** Yearly Sea Surface Temperature Anomaly from 1977 to 2016.

Year	2016	2015	2014	2013	2012	2011	2010	2009	2008	2007	2006	2005	2004	2003	2002	2001	2000	1999	1998	1997
**GLO SSTA**	0.53	0.51	0.44	0.37	0.36	0.27	0.31	0.34	0.26	0.26	0.32	0.36	0.33	0.34	0.30	0.31	0.20	0.16	0.34	0.33
**ECS SSTA**	1.30	0.60	0.53	0.80	0.39	0.25	0.39	0.57	0.91	0.73	0.70	0.65	0.65	0.71	0.68	0.39	0.70	0.59	1.40	0.52
**PS SSTA**	1.10	0.58	0.61	0.65	0.27	0.33	0.79	0.34	0.62	0.56	0.49	0.43	0.36	0.62	0.54	0.79	0.52	0.72	1.13	0.19
**SCS SSTA**	0.92	0.91	0.81	0.57	0.34	0.22	0.88	0.29	0.38	0.42	0.48	0.52	0.37	0.58	0.66	0.88	0.45	0.43	1.28	0.59
**NPO SSTA**	0.57	0.99	0.75	0.44	0.17	0.10	-0.06	0.44	0.16	0.06	0.26	0.39	0.48	0.31	0.29	-0.05	0.18	-0.12	0.12	0.49

Year	1996	1995	1994	1993	1992	1991	1990	1989	1988	1987	1986	1985	1984	1983	1982	1981	1980	1979	1978	1977

**GLO SSTA**	0.16	0.22	0.18	0.14	0.10	0.21	0.24	0.16	0.13	0.22	0.09	0.05	0.09	0.14	0.06	0.04	0.06	0.09	-0.00	0.02
**ECS SSTA**	0.60	0.33	0.63	0.07	-0.00	0.21	0.28	0.07	0.47	0.13	0.07	0.20	0.27	0.46	0.03	-0.20	0.31	-0.00	0.26	0.09
**PS SSTA**	0.35	0.61	0.28	0.22	0.05	0.24	0.16	0.33	0.56	0.42	0.20	0.02	0.26	0.41	-0.06	0.10	0.24	-0.01	0.06	0.16
**SCS SSTA**	0.47	0.44	0.10	0.41	0.22	0.28	0.24	0.22	0.59	0.82	0.10	-0.00	-0.00	0.53	0.29	0.20	0.08	0.04	-0.07	0.02
**NPO SSTA**	0.19	0.07	0.34	0.06	0.15	0.29	0.34	0.13	-0.16	0.03	0.15	-0.08	-0.02	-0.11	0.12	-0.08	0.37	-0.08	-0.12	-0.09

**Fig. 6:**
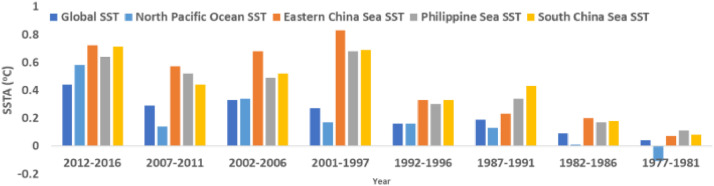
Chart of 5-yearly Sea Surface Temperature Anomaly based on data of Table 4.

**Fig. 7:**
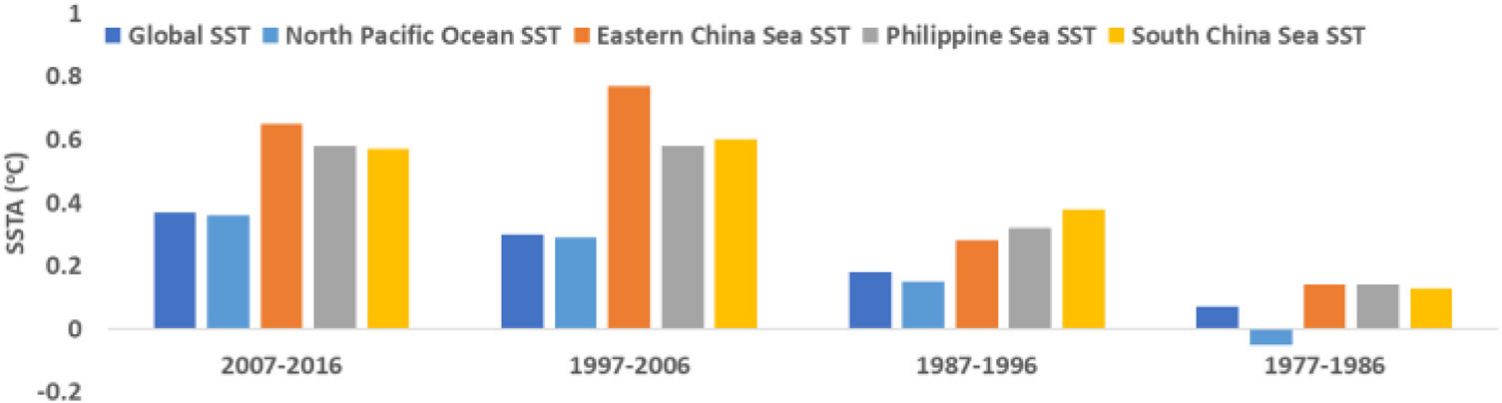
Chart of decadal Sea Surface Temperature Anomaly based on data of Table 4.

Based on Table 4 and on averaging the SSTA for every 5 years (half-decade), Fig. 6 shows the 5-yearly SSTA data demonstrating the half-decadal SSTA variations in all marginal seas in the North West Pacific along with the Global Ocean. Similarly, Fig. 7 shows the decadal SSTA data based on averaging decade-wise SSTA data of Table 4 for all marginal seas in the North West Pacific along with the Global Ocean.

Table 5 provides the 10-yearly (decadal) and typhoon season month-wise averaged SSTA data from 1977 to 2016. The table also provides month-wise SSTA data for the whole chosen 40 years. The base years are 1941-1970.

**Table 5:** Month-wise decadal averaged Sea Surface Temperature Anomaly from 1977 to 2016. Mx, Mn, and MN represent maximum, minimum, and mean SST values, respectively.

Month-wise decadal Averaged Sea Surface Temperature Anomaly (°C) during 1977-2016; Base period: 1941-1970																									
Time Period	2007-2016					1997-2006					1987-1996					1977-1986					1977-2016				
Month	ECS	PS	SCS	NPO	GLO	ECS	PS	SCS	NPO	GLO	ECS	PS	SCS	NPO	GLO	ECS	PS	SCS	NPO	GLO	ECS	PS	SCS	NPO	GLO
May	0.64	0.44	0.46	0.29	0.32	1.18	0.60	0.56	0.19	0.28	0.16	0.21	0.31	0.15	0.19	0.25	0.13	0.14	-0.09	0.08	0.56	0.35	0.37	0.14	0.22
June	0.75	0.61	0.58	0.35	0.37	0.88	0.58	0.49	0.23	0.31	0.52	0.42	0.43	0.12	0.20	0.29	0.13	0.16	-0.06	0.09	0.61	0.43	0.42	0.16	0.24
July	0.63	0.60	0.53	0.41	0.40	0.68	0.48	0.60	0.25	0.32	0.48	0.44	0.45	0.18	0.21	0.33	0.15	0.13	-0.10	0.08	0.53	0.42	0.43	0.17	0.25
August	0.48	0.61	0.55	0.40	0.43	0.53	0.43	0.54	0.25	0.34	0.16	0.34	0.41	0.08	0.20	-0.13	0.06	0.09	-0.09	0.07	0.26	0.36	0.40	0.16	0.26
September	0.36	0.66	0.67	0.40	0.40	0.31	0.55	0.62	0.30	0.31	0.02	0.32	0.41	0.13	0.17	-0.18	0.18	0.11	0.01	0.07	0.13	0.43	0.45	0.21	0.24
October	0.78	0.54	0.56	0.35	0.32	0.75	0.70	0.70	0.29	0.25	0.17	0.28	0.40	0.18	0.11	0.12	0.15	0.14	-0.01	0.03	0.45	0.42	0.45	0.20	0.18
November	0.90	0.65	0.67	0.33	0.30	0.97	0.75	0.72	0.30	0.27	0.45	0.24	0.24	0.24	0.15	0.29	0.17	0.14	0.01	0.05	0.65	0.45	0.44	0.22	0.19

All of the provided SST and SSTA data from Table 1, Table 2, Table 3, Table 4, Table 5 are crucial for all climate and typhoon-related researchers and are free to use with proper citation.

## 2 Experimental Design, Materials and Methods

### 2.1 Data selection

**Fig. 8:**
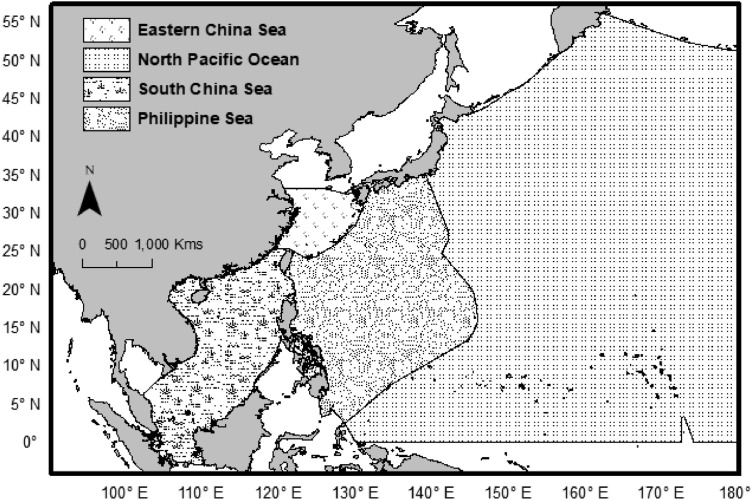
Ocean boundaries of the Eastern China Sea, Philippine Sea, South China Sea, and the North Pacific Ocean.

Although, both SST datasets used in this article provide monthly averaged SST data for the whole twelve months of a year, but to make the data useful for typhoon-related research [9], [10], [11], only the typhoon season months of May to November based data are extracted. For clarification, typhoons come in the NWP throughout the year and thus there is no exact typhoon season definition in the NWP. Several previous research in the NWP have identified May to November as the main typhoon activity period in the year and have used this timeframe for investigations [1,12], thus the presented datasets also follow the same timeframe of the year for creating the SST and SSTA related datasets. 1977 to 2016 SST data from the OI-SST V2 dataset (for 1982 to 2016) and partially from the COBE-SST V2 dataset (for 1977 to 1981) are extracted for typhoon season months to represent the recent four decades. In contrast, 1941-1970 SST data from COBE-SST V2 are extracted for typhoon season months for creating a thirty-year base period for making Sea Surface Temperature Anomalies data for the recent forty years (1977-2016). Along with the entire Global Ocean's SST data also the SST data for the North Pacific Ocean, Philippine Sea, South China sea, and Eastern China Sea are extracted, which is crucial for NWP Ocean basin-related research (Fig. 8).

For clarification, both the Philippine Sea and the North Pacific Ocean are together considered as one NWP Ocean in a few of the previous studies [12]. All the above-shown oceans’ boundaries are adopted from the International Hydrographic Organization (IHO) (1953) [13], which is provided by an online link https://www.marineregions.org/gazetteer.php?p=details&id=4332.

### 2.2 Sea surface temperature anomaly measurement technique

The Sea Surface Temperature Anomaly (SSTA) data is created, which is crucial for researchers for global warming-related research. The method is described below in five easy steps: (a) the SST data are averaged separately for each month of typhoon season (from May to November) for each successive 5-year period and a 10-year period of the chosen current period (1977-2016), (b) the SST data is averaged separately for each month from May to November for the whole forty years of the chosen current period (1977-2016) and base period (1941-1970), (c) the successive 1-year, 5-year, and 10-year SST data of the chosen current period is averaged over all seven months of typhoon season (from May to November), (d) the successive yearly, 5-yearly and 10-yearly SST raster images averaged over typhoon season are subtracted one-by-one from the thirty-years SST single raster image averaged over typhoon season to get the SSTA, and (e) the monthly separated 10-yearly and 40-yearly raster SST images of the chosen current period (1977-2016) are subtracted from the similar months of the averaged thirty-years SST images. A similar technique was adopted to create SSTA data by James et al. (2006) using the chosen current period of 2001-2005 with a base period of 1951-1980 [14].

All SST and SSTA dataset tables are put in this article as well as available on the Mendeley repository entitled “Sea Surface Temperature (SST) and SST Anomalies (SSTA) over the Last Four Decades (1977–2016) during Typhoon Season (May to November) in the entire Global Ocean, the North Pacific Ocean, Philippine Sea, South China sea, and Eastern China Sea” [15]. Note here that decadal and half-decadal SSTA data Tables can be found from the published related article to avoid resemblance [1], whereas yearly, half-yearly and decadal SST and yearly SSTA data are available in the current data paper.

## CRediT Author Statement

**Ravi Shankar Pandey:** Conceptualization, Data curation, Methodology, Writing – original draft preparation, Visualization, Investigation; **Yuei-An Liou:** Conceptualization, Data curation, Writing – original draft preparation, Writing – review & editing, Supervision.

## Declaration of Competing Interest

The authors declare that they have no known competing financial interests or personal relationships which have, or could be perceived to have, influenced the work reported in this article.

## Data Availability

Sea Surface Temperature (SST) and SST anomaly (SSTA) datasets over the Last Four Decades (1977–2016) during Typhoon Season (May to November) in the entire Global Ocean, North Pacific Ocean, Philippine (Original data) (DIB).
